# Can we predict which high myopes will develop pathological myopia?

**DOI:** 10.1111/opo.13462

**Published:** 2025-03-10

**Authors:** Quan V. Hoang, Ian Flitcroft, Nicola Rizzieri

**Affiliations:** ^1^ Department of Ophthalmology, Yong Loo Ling School of Medicine National University of Singapore Singapore Singapore; ^2^ Singapore Eye Research Institute, Duke‐NUS Medical School Singapore Singapore; ^3^ Department of Ophthalmology, College of Physicians and Surgeons Columbia University New York New York USA; ^4^ Centre for Eye Research Ireland, Environmental Sustainability and Health Institute Technological University Dublin Dublin Ireland; ^5^ Department of Ophthalmology, Children's Health Ireland at Temple Street Hospital Dublin Ireland; ^6^ University of Latvia Riga Latvia

## INTRODUCTION

**Nicola Rizzieri**

High myopia, if not properly managed, can progress to pathological myopia (PM), leading to severe ocular complications such as myopic macular degeneration and optic neuropathy, with the risk of permanent vision loss. The ability to predict which individuals with high myopia will develop PM represents a challenge in ophthalmological research, as it could enable targeted preventive interventions to slow or stop disease progression, with direct implications for visual health and global healthcare policies. This Point‐Counterpoint article focuses on the answer to this challenging question, highlighting the importance of an accurate predictive system, but also stressing the central role of effective preventive strategies.

Currently, advanced diagnostic techniques such as optical coherence tomography and artificial intelligence are making progress in providing early risk indicators. However, the issue remains a challenging goal, as the available scientific evidence is not always solid, and many of the predictions are based on non‐modifiable factors, making the clinical impact of these approaches uncertain. Here, Hoang and Flitcroft debate current strategies based on clinical biomarkers to predict the onset and progression of PM accurately, emphasising strengths and limitations of such approaches aiming to predict which individuals with high myopia will develop PM.

## POINT

**Quan V. Hoang**

It is currently possible to assess the odds of a highly myopic eye remaining stable versus developing PM based on a range of clinical baseline characteristics, but also many promising novel instruments based on biomechanics and imaging modalities, as well as machine learning approaches.

Using clinical variables, Yii et al.^1^ performed a meta‐analysis that included six longitudinal epidemiological studies with 5‐ to 18‐year follow‐ups that included two studies from Singapore,^2^, ^3^ two from Japan,^4^, ^5^ one from China^6^ and one from Germany.^7^ They determined that the older or the more myopic the eye was at baseline, then the greater the odds of new‐onset PM. Specifically, they determined the odds ratio (OR) of newly onset PM was higher with older baseline age (OR: 1.07; 95% CI:1.05–1.09; *p* < 0.001), the presence of fundus tessellation (OR: 3.02; 2.58–3.53; *p* < 0.001) and worse baseline myopia (in terms of both axial length [pooled OR: 2.03; 1.71–2.40; *p* < 0.001] and refractive error [OR: 0.77; 0.68–0.87; *p* < 0.001]). The same paper also reported that greater baseline myopia, higher education level, higher intraocular pressure and hypertension increased the risk of PM progression.

The most impactful clinical study to support the existence of clinical markers from which one can ascribe the odds of developing newly onset PM was an elegant longitudinal study from the Tokyo Medical Dental University that followed participants from childhood until adulthood when many developed PM. Specifically, Yokoi et al.^8^ followed 56 eyes in 29 Japanese children with high myopia (HM) who were <15 years old at baseline and monitored for >20 years (mean ages at the initial and follow‐up visits were 10.2 ± 3.6 years and 36.0 ± 7.6 years, respectively). Among these, 35 eyes (63%) had PM in adulthood, while 19 eyes (34%) exhibited a tessellated fundus only, 31 eyes (55%) had diffuse chorioretinal atrophy (CRA), three eyes (5%) showed patchy CRA and one eye (2%) had macular atrophy. Among these 35 new‐onset PM eyes, 29 (83%) already had limited peripapillary diffuse CRA in childhood whereas the other six had a tessellated fundus. Hence, peripapillary diffuse CRA may be used as a biomarker to predict PM onset. Guo et al.^9^ performed a clinical study to determine which clinical markers could increase the odds of developing new peripapillary diffuse CRA. They followed 274 children (mean age, refractive error and follow‐up time were 11.7 ± 2.5 years, −7.66 ± 1.87 dioptres [D] and 4.9 ± 1.2 years, respectively) and noted incident peripapillary diffuse CRA in 47 (20%) eyes. They reported that a more myopic refractive error (OR: 0.70; 95% CI: 0.54–0.92; *p* = 0.009) and greater gamma zone enlargement in the peripapillary region (OR: 8.28; 95% CI: 1.33–51.7; *p* = 0.02) increased the odds of incident peripapillary diffuse CRA.

In addition to longitudinal investigations, there are a number of cross‐sectional studies that indicate highly promising potential markers for new‐onset PM. For example, Tanaka et al.^10^ used ultra‐widefield optical coherence tomography to determine early signs of posterior staphylomas in highly myopic eyes. In seven of 55 eyes with staphyloma from 30 patients (mean age 12.3 ± 4.0 years), localised choroidal thinning was observed towards the edge of the staphyloma. The choroid returned to its usual thickness from the staphyloma edge towards the posterior pole, not only in those eyes with existing staphyloma but also in eyes prior to staphyloma development. It was suggested this incidental change in choroidal thickness could be used to predict the eventual location of staphyloma and specifically the location of staphyloma ridges.

There are also a number of recent developments with novel devices based on imaging and biomechanics that have shown clear differences between non‐myopic and myopic eyes and/or HM versus PM eyes.^11^, ^12^, ^13^, ^14^ Some of these devices show differences between non‐myopic and PM eyes (the extremes of the spectrum), with a subset of HM eyes displaying metrics more similar to non‐myopic eyes and another subset being similar to PM eyes. These metrics may serve as markers for new‐onset PM. However, given the inherent recency of such cutting edge developments, and the fact that new‐onset PM may take up to a decade to develop in HM eyes, more time and longitudinal studies are required to establish their ability to ascertain the odds of development of new‐onset PM. Using novel instruments, Liu et al.^15^ used polarisation‐sensitive optical coherence tomography to predict the progression of PM. Additionally, Ohno‐Matsui et al.^16^ described differences in the inner scleral structure between HM and PM eyes. In a separate investigation,^17^ a point‐of‐care ultrasound device was able to detect microstructural and biomechanical properties of the anterior sclera, which was used to identify the degree of myopia and specifically HM eyes with an area under the receiver‐operating characteristic curve (AUC) of 0.77. This was higher than using standard ophthalmic characteristics such as corneal curvature, central corneal thickness and anterior chamber depth (AUC = 0.63). Longitudinal studies with annual follow‐up are being conducted to determine if it can predict new‐onset PM. Liu et al.^18^ measured choroidal thickness to detect myopic macular degeneration (MMD) in HM participants using swept‐source optical coherence tomography (SS‐OCT) and found that eyes with MMD (*n* = 106, 18.7%) were older, had worse myopia in terms of both axial length and spherical equivalent and decreased choroidal thickness in all Early Treatment of Diabetic Retinopathy Study grid sectors (*p* < 0.001). Choroidal thickness showed a higher AUC of 0.907 than other characteristics (age: 0.625; axial length: 0.821 and SE: 0.821). A choroidal thickness <74 μm in the outer nasal sector demonstrated the greatest odds of detecting MMD (OR = 33.8).

As for machine learning‐based approaches, Chen et al.^19^ analysed 34 variables, including demographics, lifestyle, myopia history and SS‐OCT data to forecast the risk of MMD progression over a median follow‐up of 10.9 years, with an AUC of 0.87 achieved using the best algorithm. Variables such as thinner subfoveal choroidal thickness, longer axial length, poorer best‐corrected visual acuity, older age, female gender and shallower anterior chamber depth were the best predictors of MMD progression.

In summary, pathological myopia onset can be predicted using various clinical characteristics, clinical biomarkers derived from various imaging modalities and devices and artificial intelligence‐based approaches. A combination of these techniques could guide the timing of clinical follow‐up visits and lead to the early detection of PM, timely treatments being administered and possibly the future development of prevention strategies.

## COUNTERPOINT

**Ian Flitcroft**

As summarised so well in the point section, there is indeed some excellent work being done regarding the risk factors for the development and progression of pathological myopia (PM). Nevertheless, the first reference cited in the initial defence of this question concludes that most PM risk/prognostic factors are not supported by an adequate evidence base at present. As noted by Yii et al., the data are derived from a limited number of populations and most of these studies were exploratory in nature.^1^ Only one European study met the criteria for meta‐analysis, and this had a moderate risk of bias due to a low baseline participation rate. In addition, many potential confounding factors were not taken into consideration such as the impact of different environmental exposures or genetic factors. While it is still valid to claim that it is ‘currently possible to assess the odds of a highly myopic eye remaining stable versus developing the onset of pathologic myopia’, that statement is not the same as the question asked, namely, ‘which high myopes will develop PM?’ This question is an open‐ended one from a time perspective; in effect asking whether a given high myope (and I will come back to that point later) will develop PM during their lifetime. Answering that question requires consideration of the importance of a patient's age in the development of PM.

### The importance of age as a risk factor in PM

The time delay between the phase of myopic progression in childhood to early adulthood and the start of myopia‐associated visual impairment is over 20 years. All of the current studies looking at PM risk in a prospective manner are limited to 10 or 12 years of duration and are often flawed in design. For example, the 12‐year study of Foo et al.^3^ involved 859 subjects, but this represented only 13% of the original cohort leading to a high risk of bias. The best designed studies, with the least risk of bias, have durations of only 5 years.^6^, ^7^ Furthermore, nearly all the predictive risk factors cited in the first part of this article are non‐modifiable (e.g., age, sex and current anatomical changes in the fundus or choroid). While understanding the risk factors for PM is an interesting challenge, focussing on predictive models that rely on non‐modifiable risk factors is of limited clinical utility. Predicting which adults are at highest risk of worsening PM would be of benefit if there were interventions that could slow down such progression (i.e., secondary prevention). Currently, there are no proven interventions to achieve this. Therefore, rather than trying to predict which adults will develop PM in the next 5 or 10 years, it would be more beneficial to target the modifiable risk factors, namely refraction and axial length, early in life with active myopia management.

Can the current studies on PM risk factors help us decide who should be offered myopia management? By the time a young adult has reached high myopia, the potential onset of visually threatening complications of PM is so far into the future that it is outside the time range of all current predictive studies. The intervention window to reduce their future risk is a decade earlier, when they are progressing childhood myopes, making any predictions even more precarious. This means that we cannot predict who is at risk of PM in 20 or 30 years with enough certainty to be useful as a means of targeting treatment only to such patients. Considering that PM is only one potential complication of myopia and that other complications can occur in the range that was once considered physiological,^20^, ^21^ such a strategy would be of questionable validity, even if it were possible.

The clinical importance of PM lies in its association with visual impairment in older patients. The interaction between refractive error and age as risk factors for visual impairment and myopic macular degeneration is highly non‐linear.^22^, ^23^ The quantitative relationship between visual impairment, spherical equivalent refraction (ser) and age described by Bullimore et al.^24^ and shown in equation 1 indicates that an extra 1 D of myopia is approximately equivalent to 2 years of additional ageing (derived from the ratio of 0.122 and 0.057, which are the weights given to ser and age, respectively).

(1) $$\begin{array}{c} \text{Cumulative odds of visual impairment}\\ \;=10^{\left(0.057.\mathrm{age}-0.122.\mathrm{ser}-4.03\right)} \end{array}$$

This relationship has important implications for the question in hand. In simple terms, the length of a person's life is almost as important as their level of myopia when it comes to predicting the risk of visual impairment. The importance of age was also well demonstrated by the Singapore Epidemiology of Eye Diseases (SEED) study across all categories of myopia.^23^ Low myopes (−0.5 to −3.0 D) over 70 years of age had a higher prevalence of MMD than high myopes (≤ −6.0 D) under 50 years of age. This means that to predict a particular individual's lifetime risk of PM, we need to be able to predict the length of their life. While this can be estimated at population level, it is essentially impossible for a given individual. It also means that an individual who may be deemed to be at low risk at a given age, such as 75 years, may have a very high lifetime risk if they live into extreme old age. Due to the 30 or more year delay between the development of myopia and the onset of its pathological complications, we also need to account for future increases in live expectancy. By 2050, people are projected, on average, to life 5 years longer.^25^ The relationship between age and refractive error means that 5 years of life equates to 2.4 more dioptres of myopia. This means that that risk predictions based on data from the current generation will not be valid in the future for the young high myopes of today.

### Linkage of high myopia and pathologic myopia

Another problem I have with the question, as phrased, is the implicit assumption that only high myopes develop PM. The International Myopia Institute (IMI) definition of pathologic myopia, that is, ‘excessive axial elongation associated with myopia that leads to structural changes in the posterior segment of the eye (including posterior staphyloma, myopic maculopathy and high myopia‐associated optic neuropathy) and that can lead to loss of best corrected visual acuity’, does not include a reference to any myopic threshold.^26^ This is due to the fact that PM is not limited to high myopes. While it is certainly true that high myopes have a much higher prevalence of the typical complications of PM, such as MMD, such complications are certainly not limited to high myopes. In the SEED study, the number of patients with MMD equal to or worse than grade 2 was higher in lower myopes (−0.5 to −5.0 D, *n* = 198) than in higher myopes (≤ −5.0 D, *n* = 136).^23^ The reason for this paradoxical finding was that there were five times more lower myopes than higher myopes in this study, confirming the adage that rare complications of common conditions are more common than the common complications of rare conditions.

### Implications for future research

Fundamentally, my concern about this topic is that it emphasises the wrong challenge in PM. Being able to predict the future development of PM in an established high myope is an appealing technical challenge. With current techniques, it is a relatively simple, analytical task to derive risk models for PM from a large set of data. It is a task in which machine learning and artificial intelligence (AI) have already shown great promise. This technology is evolving at such a pace that our predictions of PM progression may become very accurate in statistical terms, but how will it impact patient outcomes? If the identified risk factors are not modifiable, then what is the value? We can warn patients that they are likely to suffer from visual impairment in the coming years, while offering no hope of changing their fate. By congratulating ourselves that we are doing a good job at identifying such risk factors with ever more sophisticated AI models, we are ignoring the elephant in the room. Namely, that we have no proven interventions that can reduce the risk of a person developing PM, other than reducing myopic progression in early life. The lack of treatments for PM, aside from those for neovascular complications which have poor long‐term outcomes,^27^ and the fact that many cases of MMD occur in lower degrees of myopia point to the importance of primary prevention and early intervention strategies for myopia. This should remain the main focus of our global efforts to manage myopia and its complications. In the field of PM, far greater emphasis needs to be placed on developing interventional strategies to slow it down, not just predict its progression.

## SUMMARY

**Nicola Rizzieri**

- The prediction of PM is of crucial importance. Clinical biomarkers such as age, refractive error and axial length can help predict the onset and progression of the disease.
- Non‐modifiable factors such as age and genetics play a crucial role in predicting PM, but modifiable factors must be considered as well.
- New technologies, such as optical coherence tomography and machine learning, may improve the prediction of PM.

## ISSUES TO BE RESOLVED

- Longitudinal studies and advanced technologies are essential to predict the onset and progression of PM accurately, but the current evidence is insufficient or unreliable. Actually, longitudinal studies do not cover a sufficient time span to accurately predict long‐term PM. They are limited to small sample sizes, are not sufficiently ethnically diverse and lack consideration for important confounding factors.
- The clinical utility of these predictions in preventing visual damage is limited by the lack of effective treatments to stop the progression of PM. Focusing on effective treatments and preventive measures may be more important than prediction itself.
- Despite emerging technologies predicting capabilities, early prevention is mandatory especially in young children. Myopia management strategies to prevent onset and slow myopia progression in children may be a more promising approach compared to predicting PM in adulthood.

## FUNDING INFORMATION

None.

## CONFLICT OF INTEREST STATEMENT

The authors declare that they have no conflicts of interest.
